# Combined bias suppression in single-arm therapy studies

**DOI:** 10.1111/j.1365-2753.2007.00903.x

**Published:** 2008-10

**Authors:** Harald J Hamre, Anja Glockmann, Gunver S Kienle, Helmut Kiene

**Affiliations:** 1 Senior Researcher, Institute for Applied Epistemology and Medical MethodologyFreiburg, Germany; 2 Medical Information Specialist, Institute for Applied Epistemology and Medical MethodologyFreiburg, Germany; 3 Director, Institute for Applied Epistemology and Medical MethodologyFreiburg, Germany

**Keywords:** bias suppression, patient dropouts, prospective studies, regression to the mean, research design, spontaneous remission

## Abstract

**Rationale, aims and objectives:**

For therapy evaluation studies, control groups are sometimes not feasible. In single-arm studies, various bias factors apart from the test therapy can affect clinical outcomes. The objective of this analysis was to improve the methods to minimize bias in single-arm studies.

**Method:**

We present a procedure for combined suppression of several bias factors, using two methods: sample restriction to patients unaffected by bias, and score adjustment. The procedure was used for a secondary analysis of disease score (doctors’ global rating, 0–10) in a cohort of patients receiving anthroposophic therapies for chronic diseases. Four bias factors were suppressed stepwise: attrition bias (by replacing missing values with the baseline value carried forward), bias from natural recovery (by sample restriction to patients with disease duration of ≥12 months), regression to the mean due to symptom-driven self-selection (by replacing baseline scores with scores three months before enrolment) and bias from adjunctive therapies (by sample restriction to patients not using adjunctive therapies).

**Results:**

In the cohort analysed, these four bias factors could together explain a maximum of 37% of the 0- to 6-month improvement of disease score.

**Conclusion:**

Combined bias suppression, using sample restriction and score adjustment, is a transparent procedure to minimize bias in single-arm therapy studies. Further applicability of the procedure should be tested in future studies.

## Introduction

In therapy evaluation studies, a number of factors apart from the test therapy can contribute to the outcome, for example, natural recovery, adjunctive therapies and observation bias. In controlled studies, the effects of such bias factors can be suppressed by subtracting the outcome of the control group from the outcome of the therapy group (with adjustment for baseline differences if the groups are not randomized). Sometimes, however, control groups are not feasible, for example, in the case of rare diseases [1], strong therapy preferences [2] or for ethical reasons [3]. Fifty years after the introduction of the randomized clinical trial [4], more than half of published studies even in front-line journals are not randomized [5] (in surgery more than 90%[6,7]), and for many therapies (e.g. drugs [8–11], surgery [1,12–14], other procedures [2,15]) the evidence base consists exclusively of studies without control groups. In reports of single-arm therapy studies, the issue of therapy effects versus bias is often only briefly mentioned, or at the best, the influence of one or two bias factors is analysed separately.

This paper contributes to the quality improvement of single-arm studies by presenting a procedure for combined suppression of four bias factors that may commonly affect outcomes: attrition bias, natural recovery, regression to the mean (RTTM) and adjunctive therapies. (The procedure does not encompass other factors such as placebo effects. However, these factors do not always have a relevant influence on clinical outcomes [16,17]. This issue will be dealt with in a separate paper.)

There are two simple and transparent methods for reliable suppression of individual bias factors without a control group. Each method has an underlying premise and can be applied if the premise is fulfilled:

1. If, as a premise, one *necessary precondition for the occurrence of a certain bias in certain patients* can be identified on account of patient characteristics, suppression of this bias is possible by *sample restriction*: the exclusion of patients with the respective characteristics from the study or the analysis, thus restricting the analysed sample to patients unaffected by this bias.
2. If, as a premise, the *maximal plausible impact of a certain bias on the outcome* can be established, suppression of this bias is possible by *score adjustment*: re-analysis of the outcome after subtracting the maximum plausible bias impact (analogous to subtracting the outcome of the control group in a controlled study).

Technically, both methods of bias suppression are one-way sensitivity analyses, assessing the ‘worst case’ of maximum plausible bias impact [18]. The objective is thus not to identify the real magnitude of bias impact (because this magnitude is often unknown), but to suppress the *maximum plausible* bias impact and subsequently analyse study outcomes under largely bias-free conditions.

For simultaneous suppression of several bias factors (technically a ‘worst case’ extreme scenario analysis [18]), a further premise is that the techniques used for suppression of each factor can be combined. The implementation of combined bias suppression is illustrated in the following.

## Methods

### Study sample and outcome measure

Combined bias suppression was used for a secondary analysis of data from the Anthroposophic Medicine Outcome Study (AMOS), a prospective multicentre cohort study of outpatients aged 1–75 years starting anthroposophic therapies (art, eurythmy exercises, rhythmical massage or medication) for various chronic diseases. Patients were enrolled from 1998 to 2005. A 2-year analysis of patients enrolled up to 31 March 2001 had shown significant improvement of disease symptoms and quality of life [19]. Most improvements occurred during the first 6 months after study enrolment, during which the anthroposophic therapies were implemented.

The present analysis concerned the 0- to 6-month change of disease score (doctor’s global assessment of disease severity, 0 = not present, 10 = worst possible, documented after 0, 6 and 12 months) and was performed on patients enrolled from 1 July 1998 to 31 March 2001 with disease score available at baseline (*n* = 887 of 898 enrolled patients). The majority of patients (88.5%, 785/887) were recruited by primary care doctors. Mean age was 35.6 years (SD 18.5); 73.1% (648/887) were women. Most frequent main diagnoses, classified by the International Classification of Diseases, Tenth Edition, were F00-F99 mental disorders (32.1%, 285/887 patients), M00-M99 musculoskeletal diseases (19.1%), J00-J99 respiratory diseases (9.0%) and G00-G99 nervous system disorders (7.0%). Mean disease duration was 6.5 years (SD 8.4).

### Data analysis

Disease score was analysed using a stepwise suppression of four bias factors: attrition bias, natural recovery, RTTM and adjunctive therapies. Each subsequent step was added to the previous steps, provided that the potential impact of the respective bias on the outcome was found to be positive.

Bias factors may have a potential positive impact on the outcome (i.e. the suppression of the respective bias reduces the magnitude of the improvement) or a potential negative impact (suppression increases the improvement). For bias factors with a potential negative impact, the ‘worst case’ is zero impact; therefore, the suppression of such biases was not included in the final analysis.

Paired samples were analysed with *t*-test, using SPSS 14.01.

### Step 1: Attrition bias

Patient attrition can bias the outcome if patients with missing outcome data are excluded from the analysis but have less favourable results than respondents. This bias can be suppressed by subtracting its maximum plausible impact on the outcome, that is, by replacing the missing values with values reflecting the maximum plausible degree to which dropouts may have less favourable results than respondents. The maximum plausible impact of attrition bias will depend on the individual study situation:

In AMOS, doctors’ 6-month follow-up documentation of disease score was available for 82.5% of patients (732/887) and missing for 17.5%. (The corresponding patient 6-month follow-up documentation was available for 91.4% (811/887) and missing for 8.6%.) Patients with and without available follow-up documentation did not differ significantly in age, sex, diagnosis, disease duration or baseline disease score. For patients with evaluable data at 6-month follow-up, disease score was improved from baseline in 83.5% of patients (611/732), unchanged in 11.6% and deteriorated in 4.9%.

The maximum plausible extent of attrition bias was assumed to be that, in patients with missing values, disease score would be unchanged from baseline. Accordingly, replacement of missing values with baseline values should fully suppress attrition bias.

*Suppression of attrition bias:* To minimize the potential for bias, missing values were replaced with the baseline value carried forward.

### Step 2: Bias from natural recovery

Natural recovery (permanent reduction or disappearance of symptoms without effective therapy) must be distinguished from RTTM because of symptom fluctuation and self-selection at symptom peaks (see Step 3). The potential for natural recovery diminishes with increasing disease duration and will eventually approach zero. According to the empirical literature, no relevant improvement will be expected in cohorts with typical AMOS diagnoses after 1 year’s duration or even earlier. Therefore, restricting the analysis to patients with disease duration of ≥12 months will suppress natural recovery bias. (We searched the literature for meta-analyses or representative studies of common AMOS diagnoses. In five diagnoses investigated, no relevant natural recovery was found beyond 2–3 months’ duration (low back pain [20], migraine [21], tension headache [21] and generalized anxiety disorder [22]) and 12 months’ duration (major depression [23–28]), respectively.)

*Suppression of bias from natural recovery:* To minimize the potential for bias, the sample was restricted to patients with disease duration of ≥12 months (75.6% of patients, 671/887).

### Step 3: Bias from regression to the mean

**Figure 1:**
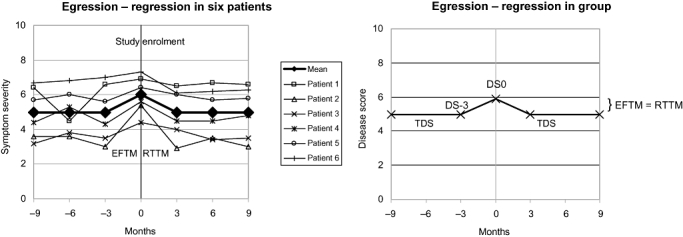
Model of egression from the mean (EFTM) and regression to the mean (RTTM) due to symptom-driven patient self-selection. True disease score (TDS) = DS-3 (disease score 3 months before study enrolment). DS0: disease score at study enrolment.

In therapy studies of symptomatic patients, RTTM can occur if symptoms fluctuate and if there is also *symptom-driven patient self-selection*, that is, if patients preferentially seek medical attention and are enrolled into the study at symptom peaks (Fig. 1). As a consequence of this self-selection, the average disease severity at study enrolment (in this sample: disease score at 0 months, DS0) will be higher than the true disease severity (true disease score). There are three important aspects of this self-selection bias:

- increase of average disease severity before study enrolment (i.e. *egression from the mean*, EFTM);
- DS0 showing higher (worse) values than true disease score; and
- return to average levels after study enrolment (i.e. RTTM).

The RTTM is a consequence of EFTM, and both will be of the same magnitude, provided that the amplitude and frequency of symptom fluctuation do not change over time (Fig. 1).

Another possible cause of RTTM in therapy studies is a *truncated patient selection*, which occurs when the inclusion criteria require a score to exceed a specific threshold, or when subgroups are analysed according to cut-off values. Truncated patient selection did not occur in the present sample, and this issue will not be further dealt with here.

Regression to the mean from self-selection at symptom peaks can be suppressed in three ways: by using a concurrent control group, by complex adjustment techniques [29–32] and by using two or more baseline scores [33–36]. For the present analysis, we used two baseline scores: DS0 and DS-3 (disease score 3 months before study enrolment, documented at study enrolment). DS-3 can be taken as an estimate of true disease score (Fig. 1). Therefore, replacement of DS0 by DS-3 will suppress RTTM bias (see Fig. 1).

*Suppression of RTTM-bias:* To minimize the potential for bias, DS0 was replaced by DS-3.

(For the present analysis of AMOS patients recruited from 1 July 1998 to 31 March 2001 (AMOS-98-01), a direct replacement with DS-3 was not possible, because study documentation of DS-3 had only been performed on AMOS patients recruited from 1 September 2004 to 31 December 2005 (AMOS-04-05). As a substitute, the difference (DS0 − DS-3) was calculated in AMOS-04-05 (average 0.43 points, *n* = 107 patients with disease duration of ≥12 months). This difference (DS0 − DS-3 = EFTM) was then subtracted from DS0 in AMOS-98-01, in order to estimate DS-3 in this sample. AMOS-98-01 and AMOS-04-05 patients fulfilled the same eligibility criteria and did not differ significantly in age, sex and main diagnosis. This necessity to impute data from other study patients is a technical limitation of the present analysis, which can be avoided in future studies.)

### Step 4: Bias from adjunctive therapies

Adjunctive therapies may affect clinical outcomes. In AMOS, adjunctive therapies were allowed, and at 6-month follow-up the patients documented their use of adjunctive therapies during the first six study months (medication use was also documented at 3-month follow-up). Because AMOS comprised a range of disorders, for which different therapies might affect outcomes, an analysis of diagnosis-related adjunctive therapies was not feasible for all diagnoses. The present analysis was therefore performed on the largest evaluable diagnostic subgroups: patients with a main diagnosis of mental, respiratory, musculoskeletal or headache disorders (67.1%, 450 of 671 patients with disease duration ≥ 12 months).

*Suppression of bias from adjunctive therapies:* To minimize the potential for bias, this sample was restricted to patients not using diagnosis-related adjunctive therapies during the first 6 months after study enrolment (54.7% of patients, 246/450). Diagnosis-related adjunctive therapies were any of the following therapies, if used for at least 1 day per month:

- mental disorders: psychotherapy (in children ergotherapy or play therapy); anti-epileptic, psycholeptic, analeptic and anti-addiction drugs (Anatomical Therapeutic Chemical Classification Index N03A, N05-06, N07B);
- respiratory disease: relevant respiratory drugs (H02, J01-02, J04-05, J07A, L03, R01, R03, R06-07) or surgery;
- musculoskeletal disease: immunosuppressive, musculoskeletal, analgesic and antidepressant drugs (L04, M01-05, M09, N02A-B, N06A); physiotherapy or relevant surgery; and
- headache disorders: analgesics, antimigraine and antidepressant drugs (C04AX01, C07AA05, C07AB02, C08CA06, C08DA01, N02, N03AG01, N06A, N07CA03).

## Results

Each of the bias suppression Steps 1–3 (suppression of attrition bias, natural recovery and RTTM) leads to a successive reduction of the 0- to 6-month improvement of disease score (Table 1). Altogether, Steps 1 + 2 + 3 reduced the average improvement by 37% (2.97 → 1.87 points).

**Table 1:** Disease score with stepwise bias suppression

				0–6 months difference		
Bias suppression steps	*n*	0 months Mean ± SD	6 months Mean ± SD	Mean	95% CI	*P*-value
*Patients with all diagnoses*						
Patients with evaluable data at 0 and 6 months	732	6.40 ± 1.76	3.43 ± 2.23	2.97	2.79–3.14	*P* < 0.001
Step 1: BVCF	887	6.38 ± 1.76	3.93 ± 2.41	2.45	2.29–2.61	*P* < 0.001
Step 1 + 2: BVCF, patients with disease duration of ≥12 months	671	6.41 ± 1.79	4.11 ± 2.37	2.30	2.12–2.49	*P* < 0.001
Final analysis *–* Step 1 + 2 + 3*:* BVCF, patients with disease duration of ≥12 months, adjustment of baseline score to suppress RTTM	671	5.98 ± 1.79	4.11 ± 2.37	1.87	1.69–2.06	*P* < 0.001
*Patients with mental, respiratory, musculoskeletal or headache disorders*						
Patients with evaluable data at 0 and 6 months	480	6.36 ± 1.71	3.23 ± 2.11	3.14	2.92–3.35	*P* < 0.001
Step 1 + 2 + 3: BVCF; Patients with disease duration of ≥12 months, adjustment of baseline score to suppress RTTM	450	6.00 ± 1.72	3.92 ± 2.33	2.08	1.85–2.31	*P* < 0.001
Step 1 + 2 + 3 + 4: BVCF; Patients with disease duration of ≥12 months, adjustment of baseline score to suppress RTTM, patients without diagnosis-related adjunctive therapies	246	5.95 ± 1.68	3.65 ± 2.28	2.30	1.99–2.61	*P* < 0.001

BVCF, missing data replaced by the baseline value carried forward.

RTTM, regression to the mean.

In patients evaluable for Step 4, that is, patients with mental, respiratory, musculoskeletal or headache disorders, Steps 1 + 2 + 3 resulted in a similar reduction of the improvement by, altogether, 34% (3.14 → 2.08 points). However, the restriction of this sample to patients not using diagnosis-related adjunctive therapies (Step 4) leads to a more pronounced improvement of disease score (2.30 points), compared with the improvement of all patients with these diagnoses (2.08 points). Thus, the potential impact of adjunctive therapy bias was not positive but negative. Accordingly, Step 4 was not included in the final analysis.

**Figure 2:**
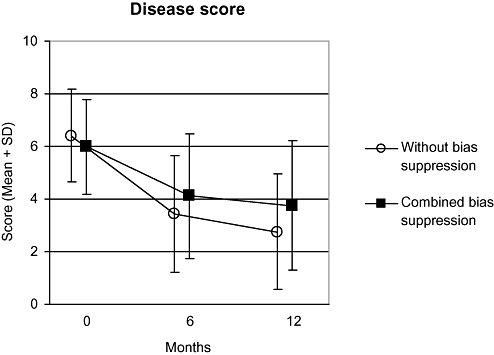
Disease score without bias suppression and with combined bias suppression. (Final analysis: suppression of bias from patient attrition, natural recovery and regression to the mean. The additional suppression of adjunctive therapy bias would not further reduce the improvement). Patients with all diagnoses.

The final analysis of disease score, after suppression of the maximum plausible positive bias impact by combining Steps 1 + 2 + 3 in patients with all diagnoses (Fig. 2), showed a significant 0- to 6-month improvement of 1.87 points (95% confidence interval 1.69–2.06 points).

## Discussion

We have presented a procedure for the combined suppression of four bias factors that may affect outcomes in single-arm studies: bias from patient attrition, natural recovery, RTTM and adjunctive therapies. The procedure combines two methods: *sample restriction* (restricting the study sample to patients unaffected by the respective bias) and *score adjustment* (re-analysis of the outcome after subtraction of the maximum plausible impact of the respective bias). The procedure was illustrated by the analysis of a study of primary care patients with chronic diseases receiving anthroposophic therapies (AMOS). After combined suppression of bias from natural recovery and adjunctive therapies (by sample restriction) and of attrition bias and RTTM (by score adjustment) it was found that these four bias factors could together explain a maximum of 37% of the 0- to 6-month improvement of disease score (0–10), with a residual significant improvement of 1.87 points.

Although many techniques for bias suppression are known [37], the simultaneous application of different techniques to suppress different bias factors within one study does not appear to be widely used. To our knowledge, the combination of sample restriction and score adjustment has not been presented previously.

The suppression techniques used here largely fulfil the premises for reliable bias suppression: The premise for *sample restriction* is that the patients potentially affected by the respective bias can be identified and excluded from the study or the analysis. Because *adjunctive therapies* can only affect patients who use them, sample restriction to non-users of diagnosis-related adjunctive therapies should reliably suppress this potential bias. Bias from *natural recovery* will be largely excluded by sample restriction to patients with disease duration of at least 1 year. The reason is that after 1 year’s duration, natural recovery of chronic disease in primary care does not generally occur. (In five AMOS indications investigated, no relevant natural recovery is found after follow-up periods ranging from 2 to 12 months.)

The premise for *score adjustment* is that the maximal plausible impact of the respective bias can be established and subtracted from the outcome. For the 6-month analysis of disease score in AMOS, the maximum plausible impact of *attrition bias* was assumed to be a no change from baseline in all patients with missing data – a clearly conservative assumption, as 83% of evaluable patients were improved from baseline. The maximum plausible impact of *RTTM bias* from symptom fluctuation and self-selection at symptom peaks was assumed to equate the full amount of symptom deterioration observed during the 3 months preceding study enrolment (DS0 − DS-3). This is probably also a conservative assumption, as some of this deterioration will not represent symptom fluctuation but permanent deterioration; for example, in major depression and in low back pain, higher symptom severity is associated with a more unfavourable prognosis [38,39].

Thus, the bias suppression techniques used in the present example were conservative, that is, aimed at protecting against false-positive results. This principle should be upheld in future studies.

The advantages of combined bias suppression as presented here are its transparency and the modular, stepwise procedure, enabling other researchers to add or delete steps as needed, or to modify steps, substituting other techniques for the suppression of individual bias factors.

As an example, to suppress bias from natural recovery, we restricted the sample according to the criterion of disease duration. For other studies, the use of this criterion may pose difficulties or be considered insufficient. However, there is a possibility of adding a second criterion: In individual patients with only moderately long prior disease duration, a short time span between beginning of treatment and clinically relevant improvement makes spontaneous improvement more unlikely than long disease duration alone. Therefore, a combined criterion (long disease duration and short time to improvement) may prove more useful than long disease duration alone.

To suppress bias from adjunctive therapies, we restricted the sample to non-users of disease-relevant therapies. In other instances, most or all patients will use adjunctive therapies, making sample restriction to non-users unfeasible. However, it may still be possible to predefine patient groups on a stable adjunctive therapy regimen (e.g. ongoing use of antidepressants or corticosteroids in stable doses), for which the addition of a new therapy may be assessed.

For minimization of EFTM/RTTM from symptom-driven patient self-selection, we replaced DS0 with DS-3. This method has two advantages. First, using DS-3 allows for a direct suppression of EFTM (which is the primary source of bias in patient self-selection), instead of suppressing RTTM (which is secondary to EFTM). Thus, no assumptions of EFTM and RTTM being equal are needed. Second, no imputing of data from reference groups is needed nor any assumption about identical score variability of study sample and reference group. Nonetheless, other researchers may prefer to use one of the many other techniques for RTTM adjustment [29–32].

The example of combined bias suppression presented here was a secondary (post hoc) analysis. In future studies all design and analysis elements of combined bias suppression can be pre-planned and incorporated into the study protocol. Thus, several limitations of the present analysis can be avoided. Regarding natural recovery, the cut-off point beyond which no relevant recovery will occur can be determined for all indications of interest (and not just for selected diagnoses, as here). Regarding RTTM, DS-3 can be documented for each analysed patient (instead of imputation of data obtained on other study patients, as here). Adjunctive therapy use can be documented by diaries or week charts (instead of retrospectively for the last 3–6 months, as here).

A technical requirement for combined bias suppression is an adequate sample size, which will depend on the magnitude of the outcome, the proportion of patients to be excluded from the analysis, the magnitude of patient dropout and so on.

Combined bias suppression has three important general limitations. First, the use of sample restriction means that a direct conclusion is possible only for the subsample unaffected by bias (in this analysis, patients with disease duration of ≥12 months not using adjunctive therapies). No direct conclusions are possible about the impact of adjunctive therapies in patients using them or about the impact of natural recovery in patients with disease duration of <12 months. Notably, this limitation of generalizability to similar patients receiving similar treatment is in principle shared by all other empirical evaluations. Second, the focus on maximum plausible bias impact means that no conclusions are possible about the minimum plausible, or the true bias impact. Thus, in the analysed sample, the four biases suppressed could together explain a maximum of 37% of the improvement. However, their true impact may have been 0% (even a negative impact is possible, as was found in the case of adjunctive therapies), but this issue was not the subject of the analysis. Third, and more important, combined bias suppression only covers the bias factors expressly suppressed – in the present example four factors – and not other confounders (e.g. patient expectations [40] or observation bias [41]) that can also affect clinical outcomes. The method of combined bias suppression should therefore be supplemented with a method for the identification of all potentially relevant bias factors and the assessment of their relevance for the respective outcome (Hamre *et al*., manuscript in preparation). Depending on study characteristics (patients, indications, treatment, outcomes, etc.), different bias factors may or may not be relevant.

## Conclusion

Combined bias suppression, using sample restriction and score adjustment, is a straightforward and transparent procedure for minimization of bias in single-arm therapy studies. The procedure should be tested in future studies.
